# ﻿Typification of the name *Ranunculusrionii* (Ranunculaceae)

**DOI:** 10.3897/phytokeys.226.103309

**Published:** 2023-05-29

**Authors:** Zdeněk Kaplan, Sébastien Bétrisey, Vincent Sonnenwyl, Jacqueline Détraz-Méroz, Gregor Kozlowski

**Affiliations:** 1 Czech Academy of Sciences, Institute of Botany, Zámek 1, 25243 Průhonice, Czech Republic Czech Academy of Sciences, Institute of Botany Průhonice Czech Republic; 2 Department of Botany, Faculty of Science, Charles University, Benátská 2, 12801 Prague, Czech Republic Charles University Prague Czech Republic; 3 Natural History Museum Fribourg (NHMF), Chemin du Musée 6, 1700 Fribourg, Switzerland Natural History Museum Fribourg Fribourg Switzerland; 4 Musée de la nature du Valais, Rue des Châteaux 14, 1950 Sion, Switzerland Musée de la nature du Valais Sion Switzerland; 5 Department of Biology and Botanical Garden, University of Fribourg, Chemin du Musée 10, 1700 Fribourg, Switzerland University of Fribourg Fribourg Switzerland

**Keywords:** Franz Joseph Lagger, history of botany, lectotype, nomenclature, Ranunculales

## Abstract

Available information on the typification of the name *Ranunculusrionii* in the literature is scarce and misleading. Previously claimed type collections indicate Lagger as the collector, but the protologue discusses only the specimens collected by Rion. Original material for the name is identified, the locality of the type collection is specified, Lagger’s way of writing herbarium labels for his type specimens is described, the history of the discovery of *R.rionii* is reviewed, and the name is lectotypified.

## ﻿Introduction

Ranunculussect.Batrachium DC. is a monophyletic group within *Ranunculus* (Hörandl and Emadzade 2012) adapted to aquatic environments and characterized by transverse ridges on the pericarp, dull white flowers and a pronounced heterophylly in some species (Cook 1963, 1966). Nonetheless, this morphological delimitation is rather weak, and these characters are not unique to this group (Hörandl et al. 2005). The section currently includes 30 recognized species widely distributed mainly in the Northern Hemisphere, with the highest diversity in Europe (Cook 1966; Wiegleb et al. 2017), and additional cryptic taxa (Prančl et al. 2018; Koutecký et al. 2022). Ranunculussect.Batrachium is taxonomically difficult mainly due to its species’ reduced morphology, extensive phenotypic plasticity, and frequent hybridization and polyploidization (Preston and Croft 1997; Wiegleb et al. 2017; Prančl et al. 2018; Koutecký et al. 2022). The diversity of this group exhibits a complex pattern, comprising well-defined diploid species, autopolyploids, allopolyploids, cryptic species, primary hybrids and introgressants (Koutecký et al. 2022).

Although two global revisions have been published since the 1960s (Cook 1966; Wiegleb et al. 2017) and several studies dealt with nomenclatural issues (e.g., Jonsell 1996; Webster 1988; Bobrov et al. 2015), full and precise typifications are available for only a part of R.sect.Batrachium names. This uncertainty in the application of names causes potential problems of nomenclatural instability. Indeed, two names have been proposed for rejection recently to avoid the replacement of well-established names by their earlier synonyms (Bartolucci et al. 2022; Kaplan et al. 2023).

The aim of this article is to clarify the history of the discovery of *R.rionii* Lagger, to describe the process of its scientific description, to correctly identify the original material and to perform the typification of this name.

## ﻿Discovery, description and typification of *R.rionii*

*Ranunculusrionii* is a diploid, homophyllous species distributed in Europe (mainly in its central and eastern parts), the Middle East and Tibet (Cook 1966; Wiegleb et al. 2017; Prančl et al. 2018). The species was described by Lagger (1848) based on the specimens collected and sent to him by Rion. The protologue indicates that Rion discovered what he believed was a new species in 1845. Lagger asked Rion to collect more specimens and send them to him for examination. Rion collected numerous specimens that were in flower and fruit in 1847. Lagger examined them and agreed that this was an undescribed species. However, in order not to publish anything prematurely and not to unnecessarily increase the number of species, Lagger consulted Godron, an author of the revision of the French R.sect.Batrachium (Godron 1840), to whom he sent his view and a specimen through the help of Buchinger. Godron agreed this was a new species related to *R.paucistamineus* Tausch and *R.drouetii* F.W.Schultz ex Godr. (both these names are now considered synonyms of *R.trichophyllus* Chaix). Encouraged by this unequivocal and definite judgment by a respected expert, Lagger decided to describe the new species. The type locality was indicated as “*In stagnis quibusdam circa Sedunum* (*Sitten*)”. Sedunum is a Latin name, while Sitten is a German name for the Swiss town of Sion, canton of Valais. The note “*in nullius alterius Batrachii consortio*” indicated that *R.rionii* was the only species of R.sect.Batrachium found at the site.

Chanoine Rion died in 1856. F. O. Wolf and R. Ritz published his botanical results posthumously in the book Guide du botaniste en Valais (Rion et al. 1872). The account titled Plantae vasculares vallesiae includes *R.rionii* with the occurrence indicated as “Fossés et étangs de Chateau-Neuf et Maladeire près Sion, juillet-septembre, R.” (“R.” stands for Rion).

Three decades later, the story of the discovery of *R.rionii* was reviewed by Wolf (1879), who translated the original German protologue to French but also provided other important information. In the museum of Sion, he discovered an original text written by Rion, which was titled “*RanunculusRionii* Lagger vel *RanunculusSedunensis* mihi”. Besides a detailed morphological analysis in French and Latin, Rion also specified the type locality as “Etang de la Maladeire près Sion, où il ne se trouve aucune autre forme.”. Wolf added a comment that by his time, the pond of the Maladeire was already dried up, and thus the type locality of *R.rionii* was destroyed. The position of the pond was at 46°13'28"N, 7°19'15"E (WGS 84), in the western part of the present-day town of Sion. The provisional Rion’s designation “*R.sedunensis*” was published in this paper merely as a synonym, was not accepted by Wolf and is therefore invalid according to the Code (Art. 36.1; Turland et al. 2018).

Available information on the typification of the name *R.rionii* in the literature is scarce. Cook (1966), in his monograph, cited the specimen “In stagnis quibusdam circa Sedunum (Sitten) Lagger.” from K as the “isotype”. This was not intended and cannot be considered as a lectotypification because Cook only indicated where the alleged duplicate of the type seen by him is preserved. Therefore, the requirements of the Code (Art. 7.11; Turland et al. 2018) were not met. Consequently, the provisions of Art. 9.10 (Turland et al. 2018) on correcting a misused term does not apply here. Wiegleb et al. (2017) cited another duplicate from this gathering as a “type” from BM. This typification was also not effective because it did not comply with Art. 7.11 (Turland et al. 2018), specifically with the requirement that on or after 1 January 2001, the typification statement must include the phrase “designated here” or an equivalent, and with Art. 9.23 (Turland et al. 2018) further requiring the use of the term “lectotypus”, its abbreviation, or its equivalent in a modern language.

Even the information on the author of the plant name, Franz Joseph Lagger, is rare. The otherwise exhaustive monograph Taxonomic Literature (Stafleu and Cowan 1979) does not include this botanist, and the databases of Harvard University Herbaria & Libraries (https://kiki.huh.harvard.edu/databases) do not provide information on the whereabouts of his types and personal herbarium as well.

Searching in herbaria yielded numerous authentic specimens that relate to *R.rionii*. The Rion personal herbarium was donated to SION in 1860 (Burnat and Fleury 1912; Praz 2013). SION preserves a gathering of *R.rionii* consisting of five sheets collected by Rion in “Etang inférieur de Maladeire. Août. 1845” and identified by him as “*R.sedunensis* mihi”. Although this is clearly the first specimen in the story of the discovery of *R.rionii*, there is no indication that this particular specimen was seen by Lagger. The provisional designation “*R.sedunensis*” given on the label is not mentioned in the protologue of *R.rionii*, while the name *R.rionii* is not given on the herbarium label. That is why this collection cannot be unequivocally considered as original material in the sense of the Code (Art. 9.4; Turland et al. 2018), as it is unclear if it was available to Lagger prior to, or at the time of, preparation of the description validating the name *R.rionii*.

Specimens labelled “*RanunculusRionii* Lagger! Vallesia pr. Sitten in fossis. Jul.-Sept. Lagger.”, “*RanunculusRionii* Lagg. Prope Sedunum in Vallesia. Dr. Lagger.” or “*RanunculusRionii* mihi. Près de Sion, Valais. Dr. Lagger.”, some of them including the year 1848, have been discovered in B, BM, GAP, JE, K, LAU, LY, LYJB, NCY, NHMF, P, PRC, S, W, WU, and ZT. Undoubtedly, duplicates of these specimens were also distributed to other herbaria around the world. The existence of these numerous collections is rather surprising because the protologue does not indicate that Lagger himself would have visited the type locality and collected any specimens. On the contrary, the protologue clearly states that Rion collected the rich gathering provided to Lagger. These collections are obviously authentic, but are they part of the original material?

Physicist and botanist Franz Joseph Lagger’s personal herbarium is currently incorporated in NHMF (Kozlowski 2001). It comprises around 18,000 specimens from all over the world. The entire Lagger herbarium is now being reviewed and digitized (Swisscollnet 2023). The examination of his collections and comparative analysis of the label records show that from the present-day point of view, Lagger was not very careful in providing precise information on the collector and collection date when writing herbarium labels. He usually wrote the year of publication of the name that he published on the type specimens instead of the actual collection date (even in his own collections, only about 10% of specimens are dated) and usually also omitted the name of the actual collector of the specimens received from other botanists. These findings correspond well with the label data in the abundant Lagger’s collection of *R.rionii*, individual specimens of which are now found in various herbaria. Although only the name “Lagger”, and sometimes the year “1848”, is given on the labels, this may well be the gathering cited in the protologue that was actually collected by Rion in 1847 and sent to Lagger on his request. In this case, all specimens from this collection would be syntypes of *R.rionii*.

**Figure 1.**
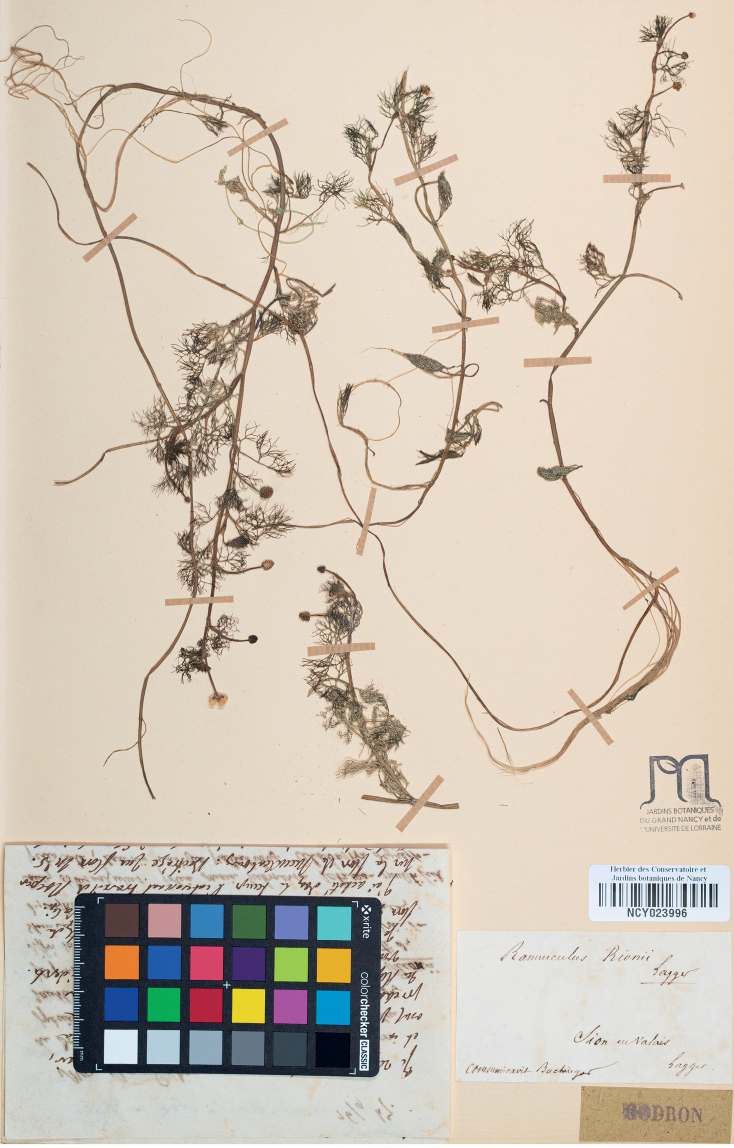
The lectotype of *Ranunculusrionii* Lagger (NCY barcode NCY023996).

**Figure 2.**
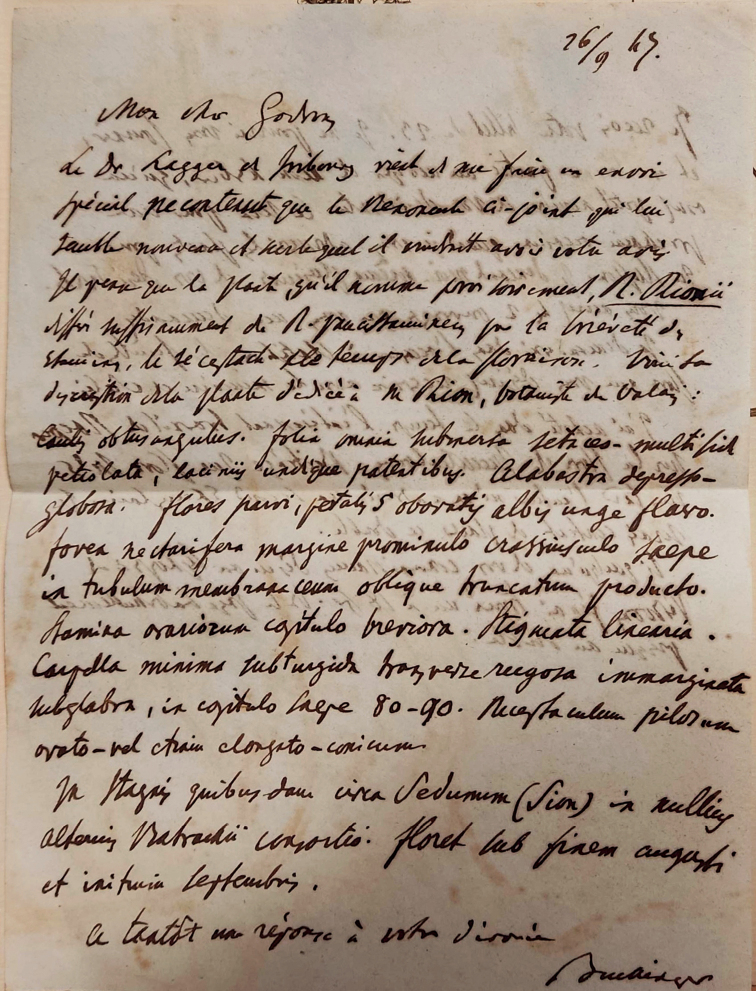
A letter from Buchinger to Godron on Rion’s discovery of a new species and with Lagger’s request on the opinion of his herbarium specimen provisionally designated as *Ranunculusrionii*.

The most important clue to the identification of the type and the elucidation of the story was the discovery of an authentic specimen in NCY (Fig. 1). This was collected at the type locality of *R.rionii*, the herbarium label bears the name *R.rionii* and the name of Lagger (as the other collections discussed above), but most importantly, it tracks the transfer of this specimen from Lagger through Buchinger to Godron, as the label shows a note “communicavit Buchinger” and the specimen is accompanied by the original letter from Buchinger to Godron, dated 26 September 1847 (Fig. 2). The letter contains Lagger’s request on the opinion on the enclosed specimen of his *R.rionii*. In addition, it includes the description and type citation (see below), exactly in the form that later appeared in the protologue. The fact that Lagger asked Buchinger to facilitate the examination of the specimen of *R.rionii* by Godron is explicitly described in the protologue as well. This specimen is, therefore, an unequivocal original material and the best candidate for the typification of *R.rionii*. The other parts of the collection with herbarium labels written by Lagger, distributed by him to various botanists and institutions, are considered as its duplicates.

The nomenclature of *R.rionii* is summarized below:

***Ranunculusrionii*** Lagger in Flora 31: 49. 1848. **Lectotype** (designated here): Switzerland, [herbarium label:] “RanunculusRionii Lagger / Sion en Valais / Lagger. / communicavit Buchinger”, [enclosed letter:] “… In stagnis quibusdam circa Sedunum (Sitten) in nullius alterius Batrachii consortio, floret sub finem Augusti et initium Septembris.”, [leg. *C. Rion s.n.*] (NCY barcode NCY023996!, Fig. 1; isolectotypes [with variations in label data]: B barcode B101112935!, BM barcode BM000613872!, GAP barcode GAP069572!, JE barcode JE00021497!, K barcodes K000675432!, K000675434!, K000675435!, K000675436!, LAU n.v., LY barcodes LY0052072!, LY0675158!, LYJB barcode LYJB045434!, NHMF barcode GBIFCH05028535!, P barcodes P02558430!, P02389952!, PRC s. no.!, S no. S08-154, W n.v., WU no. 039949!, 039950!, ZT barcode ZT-00176078!).

– *Ranunculussedunensis* Rion ex F.O.Wolf, Bull. Murith. Soc. Valais. Sci. Nat. 7–8: 38. 1879, nom. inval. [Turland et al. 2018, Art. 36.1]. Authentic specimen: Switzerland, [label 1:] “R.sedunensis mihi / … / Etang inférieur de Maladeire. août. 1845.”, [label 2:] “Herbarium Rion” (SION barcode SION000399!).
